# Active Potentiometry for Dissolved Oxygen Monitoring with Platinum Electrodes

**DOI:** 10.3390/s18082404

**Published:** 2018-07-24

**Authors:** Peter Zimmermann, Andreas Weltin, Gerald A. Urban, Jochen Kieninger

**Affiliations:** Laboratory for Sensors, IMTEK—Department of Microsystems Engineering, University of Freiburg, Georges-Köhler Allee 103, D-79110 Freiburg, Germany; peter.zimmermann@imtek.de (P.Z.); weltin@imtek.de (A.W.); urban@imtek.de (G.A.U.)

**Keywords:** oxygen sensor, platinum, potentiometry, active potentiometry, mixed potential

## Abstract

Potentiometric oxygen monitoring using platinum as the electrode material was enabled by the combination of conventional potentiometry with active prepolarization protocols, what we call *active potentiometry*. The obtained logarithmic transfer function is well-suited for the measurement of dissolved oxygen in biomedical applications, as the physiological oxygen concentration typically varies over several decades. We describe the application of active potentiometry in phosphate buffered salt solution at different pH and ion strength. Sensitivity was in the range of 60 mV/dec oxygen concentration; the transfer function deviated from logarithmic behavior for smaller oxygen concentration and higher ion strength of the electrolyte. Long-term stability was demonstrated for 60 h. Based on these measurement results and additional cyclic voltammetry investigations a model is discussed to explain the potential forming mechanism. The described method of active potentiometry is applicable to many different potentiometric sensors possibly enhancing sensitivity or selectivity for a specific parameter.

## 1. Introduction

Oxygen concentration is an important parameter for many physiological processes. Monitoring the dissolved oxygen is required for research in cell culture as well as in the in vivo situation. In cancer research for example, the relevant concentration range of oxygen covers several orders of magnitude [1]. For the monitoring of cellular oxygenation, both electrochemical and optical oxygen sensor principles are used [2]. The application of optical oxygen sensors is tempting due to the non-consuming sensor principle, but it is especially difficult for low oxygen concentration due to the reciprocal transfer function (*Stern-Volmer* behavior) of sensors using fluorescence quenching with both intensity and fluorescence decay time as readout [3]. Electrochemical oxygen sensors for dissolved oxygen are mainly based on amperometry; in contrast, sensors for oxygen in the gas phase often rely on potentiometry.

In amperometry, the oxygen molecules are reduced at an appropriately polarized working electrode causing a current proportional to the amount of reduced oxygen molecules. Typical sensor arrangements are operated in diffusion limitation leading to a linear concentration gradient of the molecular oxygen between the electrode surface and the bulk outside the sensor resulting in a linear relationship between the oxygen concentration and the reduction current. The two major disadvantages of this approach are the inherent analyte consumption as well as the non-equilibrium situation, which by principle never can reflect the undisturbed oxygen profile. The usage of chronoamperometric protocols comprising a long off-time reduces the analyte consumption and allows oxygen monitoring in cell cultures [4,5], but still inadequately covers the physiologically relevant concentration range due to the linear sensor response.

Thus, a logarithmic transfer function as can be found in potentiometry would be favorable for the dissolved oxygen measurement. Here, the open circuit potential (OCP) is measured in electrochemical equilibrium. According to the *Nernst* equation (Equation (1)), a logarithmic dependency on the oxygen concentration can be observed.

(1) $$E=E^{0}+2.3\frac{RT}{nF}\log c$$

*E* is the measured cell potential, $E^{0}$ the standard potential of the reaction, *n* the number of transferred electrons and *c* the dissolved oxygen concentration approximating its activity. *R* is the molar gas constant, *T* the absolute temperature, and *F* the Faraday constant. Consequently, the slope at room temperature is 59 mV/decade concentration change in case of a 1-electron process and 15 mV/dec in case of a 4-electron process.

Additionally, a potentiometric sensor provides a direct measurement of the oxygen concentration, independent of the diffusion situation inside the sensor and the analyte. Different potentiometric sensors for dissolved oxygen have been realized based on sodium tungsten bronzes [6,7], antimony [8], copper [9,10], cobalt [11,12], and ruthenium oxide [13] as material for the indicator electrode. Among them, most promising is the usage of copper and cobalt electrodes because of their applicability in neutral pH media and up to air-saturated oxygen concentrations as well as the availability of these metals in microfabrication technology. The potential is established as a mixed potential based on oxygen reduction and the oxidative dissolution of the metal.

However, in contrast to most of the previously suggested materials, platinum as a well-established electrode material is often used in both, macroscopic sensors and in thin-film technology. Its catalytic properties make it attractive as working electrode material in amperometric sensors. It would be favorable to use the same material for a potentiometric sensor and thereby enable simple integration of amperometric and potentiometric sensors together or even use the very same electrode for both types of measurement.

The excellent catalytic properties of platinum are simultaneously a major drawback. While it strongly catalyzes the oxygen reduction, additionally the formation of platinum oxide and subsequently passivation of the electrode is possible. The reaction of molecular oxygen at platinum is generally agreed to follow the 4-electron pathway:

(2) $$O_{2}+4H^{+}+4e^{-}⇌2H_{2}O\;E^{0}=1.23V$$

The platinum oxide layer can be formed at the electrode surface for potentials larger than 0.85 V_RHE_ [14]. The process steps can be summarized as shown in Equation (3) with a growth kinetic proportional to the logarithm of time until the formation of one monolayer (ML), which is equivalent to a charge of 440 μC/cm^2^ [15].

(3) $$Pt+H_{2}O\to PtO+2H^{+}+2e^{-}\;E>0.85V$$

Several works describe the application of platinum in potentiometric measurement of dissolved oxygen. Platinum electrodes coated with a Co(II)-tetren-doped polymer film were described in [9]. The possibility to directly apply platinum in a potentiometric oxygen sensor was identified to depend on the surface treatment, such as polishing before the measurement [16]. It was observed that oxidized platinum surfaces show no oxygen sensitivity, while reduced platinum surfaces results in reversible, potentiometric response which decrease over time [9]. However, in equilibrium the platinum electrode is passivated by an oxidized surface.

In this work, we introduce a novel procedure, which we call *active potentiometry*, to minimize the influence of the platinum oxide. Prior to potentiometric measurements in (pseudo-)equilibrium, the electrode is polarized at a defined potential to determine the surface state, e.g., whether a platinum oxide layer is formed or a bare platinum surface exists. Evaluation of the subsequent equilibrium phase allows the application of this method as sensor principle for the measurement of dissolved oxygen. Based on the active potentiometry measurement results as well as cyclic voltammetry investigations, a model for the potential forming mechanism was postulated which is able to explain the influence of the prepolarization on and the time dependence of the potentiometric measurement phase.

## 2. Material and Methods

### 2.1. Reagents and Instrumentation

Standard electrolyte in this work was phosphate buffered saline (PBS) with a pH value around neutrality. If not mentioned explicitly, the PBS consisted of 85.2 mM Na_2_HPO_4_, 14.8 mM NaH_2_PO_4_ and 100 mM NaCl in water, resulting in pH 7.4. Various pH values were achieved by mixing an aqueous solution of 100 mM Na_2_HPO_4_ and 100 mM NaCl with a solution consisting of 100 mM NaH_2_PO_4_ and 100 mM NaCl in different ratios. The influence of ion strength was investigated by diluting 10 times the standard PBS with water resulting in pH 8.2.

The measurement setup consisted of a 2 mm platinum disk electrode (6.1204.190, Metrohm AG, Herisau, Switzerland) as working electrode, a Ag/AgCl reference electrode with 3 M KCl inner filling (6.0726.100, Metrohm AG, Switzerland), and a rod-like platinum counter electrode. All measurements were done using the potentiostat CompactStat (IVIUM Technologies B.V., Eindhoven, The Netherlands) along with the software IviumSoft for the implementation of combined active polarization and potentiometric measurements. All potentials apart from the measurement of the pH-influence are reported in terms of the Reversible Hydrogen Electrode (RHE) scale. The potential of the RHE can be calculated based on the Normal Hydrogen Electrode (NHE) potential by addition of 2.3·RT/F= 59 mV at room temperature per pH unit. Potentials vs. RHE were obtained by adding 0.207 V (standard potential of Ag/AgCl at 25 ${}^{\circ }C$ in 3 M KCl) and 0.438 V (reflecting pH 7.4 at 25 ${}^{\circ }C$) to the potential according to the used reference electrode.

Different dissolved oxygen concentrations were adjusted by gas mixtures based on nitrogen gas and compressed air. The gas compositions were varied by the gas mixing station IL-GMix41 (HiTecZang, Herzogenrath, Germany). From the volume fraction of oxygen in the gas phase the concentration of dissolved oxygen in PBS was calculated using a Henry’s law constant of 12.47 μM/kPa [17] and taking into account the salting-out effect due to the buffer according to [18]. The oxygen concentration was decreased accordingly by a factor 0.891 in case of 0.1 M PBS and a factor 0.989 for the 10-times diluted PBS. All experiments were done at room temperature ((25±2) °C).

### 2.2. Active Potentiometry

**Figure 1:**
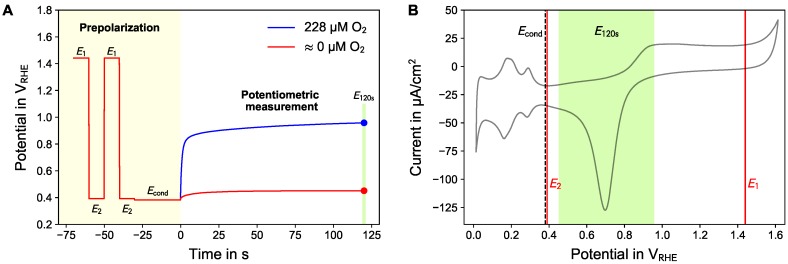
(**A**) One cycle of the *active potentiometry* comprising a prepolarization phase (marked, left region) and the potentiometric measurement phase (unmarked, right region). The results shown in this figure were obtained with $E_{\mathrm{cond}}=$ 0.38 V_RHE_ resulting in good oxygen sensitivity during the potentiometric phase. The potentiometric phase lasts for 120 s in which the measurement value is taken at its end ($E_{120s}$) providing one data point per repetition. The blue curve was obtained in air-saturated and the red curve in nitrogen-flushed PBS. (**B**) Overlay of the potentials from A over a cyclic voltammogram of platinum in air-saturated PBS to illustrate the different potential regions.

The principle of *active potentiometry* is understood as treatment of the electrode by an active prepolarization and a subsequent phase of potentiometric (current-less) measurement. In each cycle, the electrodes were conditioned and measured using the following procedure (see Figure 1):

1. To obtain a clean electrode surface, the electrode was polarized twice to $E_{1}=$ 1.44 V_RHE_ (volts with respect to reversible hydrogen electrode) for 10 s to form a platinum oxide layer, followed by $E_{2}=$ 0.39 V_RHE_ for 10 s, at which the just formed oxide is reduced back to bare platinum.
2. The cleaning procedure was followed by polarization to a conditioning potential $E_{\mathrm{cond}}$ determining the status of the electrode surface (bare platinum or platinum/platinum oxide electrode) at which the open cell phase starts. This potential was altered between 0.04 V_RHE_ and 1.29 V_RHE_ to investigate the optimal working point (Section 3.2) and for all further measurements fixed to 0.38 V_RHE_ for 30 s.
3. The preconditioning was followed by an open cell phase (potentiometric measurement) for 120 s or 360 s. The measurement value was taken at the end of this phase ($E_{120}$ or $E_{360}$).

### 2.3. Experimental Procedure

#### 2.3.1. Cyclic Voltammetry

Steady-state curves were recorded in air-saturated and nitrogen-flushed 0.1 M PBS. A slow scan rate of 20 mV/s was chosen in order to emphasize the current due to the oxygen reduction in comparison to the currents from the surface processes.

#### 2.3.2. Working Point of Potentiometry

The working (starting) point for the potentiometric phase is determined by the prepolarization potential $E_{\mathrm{cond}}$, which was varied within the limits of gas formation (0.04 V_RHE_ to 1.29 V_RHE_). For each $E_{\mathrm{cond}}$ the OCP was measured for 120 s. Measurements were done in air-saturated and nitrogen flushed 0.1 M PBS.

#### 2.3.3. Oxygen Concentration Measurements

To adjust the oxygen concentration, the electrolyte was flushed with different gas mixtures of nitrogen and compressed air. The oxygen fraction was set to 21.0%, 10.5%, 5.2%, 2.1%, 1.0% and 0%. The OCP measurement time for each cycle was set to 120 s. To measure low oxygen concentrations, the electrolyte was flushed with gas mixtures resulting in oxygen fraction of 2.0%, 1.1%, 0.54%, 0.22%, 0.11%, 0.06%, 0.03%, and 0.02%. The OCP measurement time for each cycle was set to 360 s.

#### 2.3.4. Influence of the Ion Strength of the Electrolyte

Influence of ion strength was investigated by standard 0.1 M PBS and 0.01 M PBS prepared by diluting 0.1 M PBS with water in the ratio 1:10. The dilution caused an increase from pH 7.4 to pH 8.2, which was compensated by adjusting the prepolarization potentials in case of the diluted solution. Adjustment was done according to cyclic voltammograms targeting the phenomenologically same regions. The potentials in the diluted PBS were $E_{1}=$ 1.46 V_RHE_, $E_{2}=$ 0.47 V_RHE_, and $E_{\mathrm{cond}}=$ 0.46 V_RHE_. Oxygen concentration was adjusted by flushing the solution with different gas mixtures of nitrogen and compressed air. The oxygen fraction was 22.0%, 1.1%, 0.54%, 0.22%, 0.11%, 0.06%, 0.03%, and 0.02%.

#### 2.3.5. Influence of the pH of the Electrolyte

The pH dependency of the OCP was examined in buffer solution by mixing solution A and B (see Section 2.1). The tested pH values were pH 5.59, pH 6.22, pH 6.66, pH 7.10, pH 7.53, and pH 7.98. For each solution, the prepolarization potential was adjusted according to cyclic voltammograms targeting the phenomenologically same region. For each pH value 10 active potentiometry cycles were measured.

## 3. Results and Discussion

### 3.1. Cyclic Voltammetry

**Figure 2:**
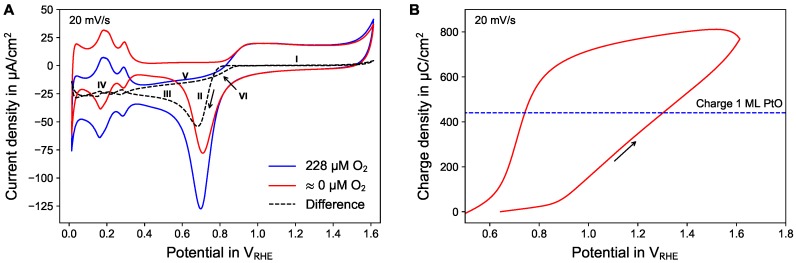
(**A**) Cyclic voltammograms (CV) of platinum in air-saturated (dashed curve) and nitrogen flushed (dotted curve) in 0.1 M PBS. The difference signal (solid curve) is assumed to represent mainly the oxygen reduction process at platinum. (**B**) Charge (integral of the current in A) for the nitrogen flushed CV in the region of platinum oxide formation. The dashed line represents the charge needed to form 1 monolayer (ML) of PtO.

Steady-state cyclic voltammograms were recorded in air-saturated and nitrogen flushed electrolyte, see Figure 2A. The difference between the two curves (dashed line) is attributed to the oxygen reduction process only. In the forward scan, starting at a potential marked around 0.8 V_RHE_, no oxygen reduction was observable. We assume this is due to the presence of PtO at the electrode (region I). The small increase of the difference for potentials larger than 1.5 V_RHE_ is interpreted as artifact due to minor differences in the current during molecular oxygen production.

After the upper turning point, in the reverse scan, it took until region II before an oxygen reduction current became visible. At this potential a substantial amount of the electrode surface was reduced back to bare platinum. Following the high oxygen reduction signal at the beginning, the current went back to smaller absolute values due to the build-up diffusion profile towards the electrode (region III). The fluctuations in region IV are not considered because of the high sensitivity of the hydrogen adsorption/desorption process to contaminations as well as minor differences in the current for the molecular hydrogen production.

The decrease of the oxygen reduction in region V is attributed to further build-up of the concentration gradient. In contrast, in region VI the decrease of the oxygen reduction was observed to show a much steeper slope. It is assumed that already some catalytically active sites of the platinum surface became blocked due to chemisorbed oxygen atoms, following the interpretation of the platinum oxide formation in this potential range as a two-step process based on discharge of water/chemisorption of oxygen followed by the formation of a quasi-3D lattice [15].

Two important conclusions for the further discussion can be drawn from the cyclic voltammograms:

1. The oxygen reduction process is inhibited by the presence of platinum oxide on the electrode (passivation). This effect occurs far away from the equilibrium potential $E^{0}$ for the molecular oxygen reduction/formation (Equation (2)), which is higher than that of the platinum oxide reduction formation (Equation (3)).
2. The amount of charge contributing to the formation of platinum oxide is less than 880 μC/cm^2^ (see Figure 2B) within the water window, which suggests that overall less than 2 ML of PtO are formed (1 ML PtO corresponds to a charge of 440 μC/cm^2^) [15]). The potential at which 1 ML PtO is formed was found to be 1.30 V_RHE_ at a scanrate of 20 mV/s. For stationary conditions, it is assumed that the potential corresponding to 1 ML PtO can be slightly lower.

### 3.2. Working Point of Potentiometry

The working point for the potentiometric measurement is defined by the last step of the prepolarization phase, the conditioning potential $E_{\mathrm{cond}}$. To find the optimal working point, cycles with different values of $E_{\mathrm{cond}}$ were applied in air-saturated and nitrogen flushed electrolyte.

**Figure 3:**
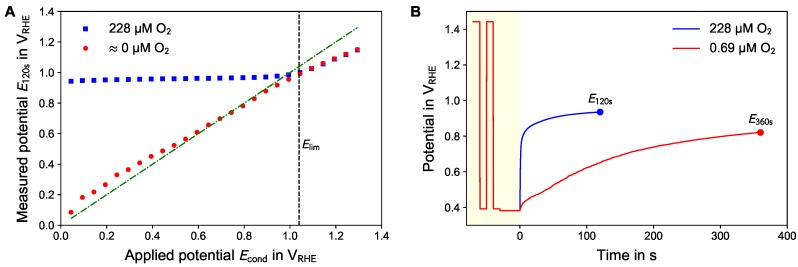
(**A**) The measured potential $E_{120s}$ as function of the the conditioning potential $E_{\mathrm{cond}}$ at the end of the prepolarization phase. Lower $E_{\mathrm{cond}}$ increase the difference between measurement in air-saturated and nitrogen-flushed electrolyte. For $E_{\mathrm{cond}}>E_{\lim}$ the measured potential did not show any oxygen dependency. (**B**) Transient behavior of the measured potential. For lower oxygen concentration (red curve) a prolonged OCP measurement phase is required.

Figure 3A shows the OCP after 120 s ($E_{120s}$) for different conditioning potentials $E_{\mathrm{cond}}$. In case of air-saturated electrolyte the $E_{120s}$ values are nearly constant around 0.95 V_RHE_ for any conditioning with $E_{\mathrm{cond}}<E_{\lim}=$ 1.04 V_RHE_. The OCP values of the nitrogen flushed electrolyte were nearly the same as $E_{\mathrm{cond}}$ for any conditioning with $E_{\mathrm{cond}}<E_{\lim}$. For any prepolarization with $E_{\mathrm{cond}}>E_{\lim}$ the resulting OCP values are independent of the oxygen concentration. In this case, the OCP values were slightly decreasing during the potentiometric phase resulting in $E_{120s}<E_{\mathrm{cond}}$.

It is assumed that the observable OCP is a mixed potential comprising oxygen reduction and platinum oxide formation. In case of sufficient oxygen concentration, the oxygen reduction and oxide formation takes place until the electrode is covered with platinum oxide.

In the absence of oxygen, the OCP of the electrode remains nearly at the previously applied potential $E_{\mathrm{cond}}$. While the platinum oxide formation process could in principal occur, the counter process (oxygen reduction) is missing and the charge cannot be balanced locally at the electrode. The electrode with its double layer acts as a capacitor keeping its voltage in absence of a charging/discharging current. Only through remaining molecular oxygen, parasitic redox processes or current flow into the high impedance input of the potentiostat, the oxide layer could be formed and the double layer capacity could be discharged, and thus the potential could increase. This does not occur within reasonable duration of the OCP measurement phase.

These results confirm the interpretation of the data shown in Figure 2A. The difference signal in the CV goes back to zero (region VI) around the same potential of 0.95 V_RHE_ which can be observed in case of air-saturation for $E_{\mathrm{cond}}<E_{\lim}$. We speculate that for longer waiting time or higher oxygen concentration this potential would approach $E_{\lim}$. This steady-state value can be associated with nearly full coverage of the electrode by a monolayer of PtO. Comparing the value of $E_{\lim}$ with Figure 2B, a higher value associated with 1 ML of PtO of 1.30 V_RHE_ was found. We speculate that all three values refer to the same electrode status and should approach the value of $E_{\lim}$ for infinite waiting time during the potentiometric phase or infinitely slow scanrate.

In case of the absence of dissolved oxygen $E_{120s}$ is roughly the same as $E_{\mathrm{cond}}$ because of the absence of the oxygen reduction as counter process to the platinum oxide formation (compare the doubled-dashed line in Figure 3A representing unity slope). In contrast, for $E_{\mathrm{cond}}>E_{\lim}$ the oxygen reduction cannot occur because of the passivation of the electrode by PtO, although the equilibrium potential of the oxygen formation/reduction reaction (Equation (2)) would still allow oxygen reduction.

The working point of the active potentiometry is defined by $E_{\mathrm{cond}}$. The maximal sensitivity can be reached if the difference between the values in air-saturated and nitrogen flushed electrolyte is highest. Another aspect is that the conditioning should not cause any secondary effect and thus should be adjusted at least above the potential where hydrogen adsorption at platinum occurs (as can be deduced from Figure 2A). Therefore, $E_{\mathrm{cond}}=$ 0.39 V_RHE_ was chosen for all further experiments.

Figure 3B shows the comparison of the trace of active potentiometric measurements in electrolytes with high ( 228 μM) and low ( 0.69 μM) dissolved oxygen concentration. For low oxygen concentration the slopes during the potentiometric phase were lower compared to the situation in air-saturated solution. Additionally, reading the potential at 120 s results in less reproducible measurements results compared to with higher oxygen concentration. Depending on the desired measurement range a duration of the OCP measurement phase of either 120 s or 360 s was used.

### 3.3. Oxygen Concentration Measurements

**Figure 4:**
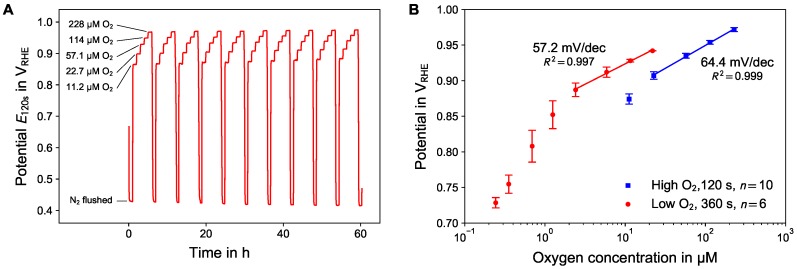
(**A**) Trace of active potentiometry measurements of 10 consecutive concentration ramps with higher oxygen concentrations. (**B**) Calibration curves for the concentrations ramp with higher and lower oxygen concentration range. In case of lower oxygen concentrations (red disks) the OCP phase was prolonged to 360 s.

Sensitivity and stability was examined measuring with repetitive oxygen concentration ramps following after purging the system with nitrogen. Figure 4A shows the trace of active potentiometry measurements of 10 consecutive concentration ramps with higher oxygen concentrations (> 10 μM) and a duration of 120 s for the potentiometric phase.

Evaluating the data, a positive drift rate of 3.1 mV/day in case of air-saturated and negative drift rate of −2.6 mV/day in case of nitrogen flushed electrolyte was found over a period of 60 h. The drifts were equally distributed over the different concentration ramps. We assume that the extensive cycling during the prepolarization ($E_{1}$ vs. $E_{2}$) continuously changed the morphology or crystallinity of the electrode surface.

Figure 4B summarizes the calibration curves for higher (10 to 200 μM) and lower (0.2 to 20 μM) dissolved oxygen concentrations. The calibration curve for high oxygen concentration shows a logarithmic transfer function with a slope of 64.4 mV/dec dissolved oxygen concentration (solid line). The calibration curve for the low oxygen concentrations was recorded using a prolonged duration of the OCP measurement phase of 360 s. The resulting curve shows a linear region in the logarithmic plot, with a slope of 57.2 mV/dec for the higher oxygen concentrations.

The constant slopes in the logarithmic calibration plot suggest a relationship according to the Nernst equation (Equation (1)). However, in case of platinum the oxygen reduction follows the 4-electron process (Equation (2)) which would lead to a sensitivity of 15 mV/dec at room temperature opposing the experimental data. While the Nernst equation describes a single, reversible redox process, we assume a mixed potential situation consisting of the oxygen reduction and PtO formation reactions as potential forming mechanism as described in Section 3.2.

In case of very low concentrations (less than 1 μM) the obtained $E_{360s}$ values deviate from the logarithmic transfer function. It is assumed that this deviation can be attributed to the onset of diffusion limitation at very low oxygen concentrations. Accordingly, the corresponding logarithmic dependency on the concentration is not valid any more. In the most extreme case, complete diffusion limitation would result in a linear transfer function. However, this would only occur for extremely low oxygen concentrations, as the overall charge available during the potential forming mechanism is limited to 440 μC/cm^2^ by the charge required for 1 ML PtO.

### 3.4. Influence of Ion Strength of the Electrolyte

**Figure 5:**
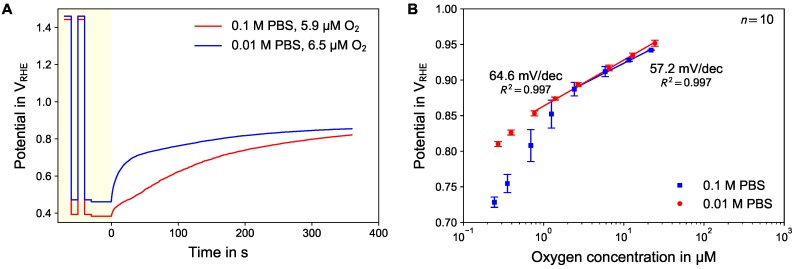
Influence of the ion-strength of the electrolyte: Comparison of time trace (**A**) for concentration with same oxygen fraction in gas phase and calibration curves (**B**) using a duration of 360 s for the OCP phase.

In Figure 5 the results of the investigation of the influence of the ion strength of the electrolyte are summarized. Figure 5A shows one active potentiometry cycle in 0.1 and 0.01 M PBS for a dissolved oxygen concentration with 0.54% volume fraction of oxygen in the gas phase. In 0.1 M PBS this corresponds to 5.9 μM in 0.01 M PBS to 6.5 μM dissolved oxygen concentration due to different salting-out coefficients. The curve in diluted electrolyte reaches a quasi-stationary state during the OCP phase in shorter time.

Figure 5B displays the calibration curves as mean value and standard deviation of the $E_{360s}$ values for both electrolyte concentrations. The calibration curve in 0.01 M PBS follows logarithmic behavior with a slope of 64.6 mV/decade dissolved oxygen concentration. In contrast, the calibration curve in 0.1 M PBS shows logarithmic behavior for higher concentration only, with a slope of 57.2 mV/decade, while for lower oxygen concentration the measured potential deviates towards lower numbers similar to the results shown in Figure 4B.

Following our previous interpretation that the charge of the double layer capacity matters in case of no or low oxygen reduction current (see Section 3.3), charge is needed to charge up the double capacity in addition to the charge for the formation of PtO. The double layer capacity itself is a function of the ion strength. We therefore speculate that for low oxygen concentration not only the onset of the diffusion limitation for the oxygen reduction, but also the influence of the charging of the double layer capacity contributes to the limiting current. Both the observation of different slopes respectively different kinetics for the two tested ion strengths (Figure 5A) and stronger deviation from linear behavior (Figure 5B) for higher ion strength support this interpretation.

In situations allowing for the adjustment of the ion strength it is recommended to use a lower ion strength as it provides better conditions for the fast potentiometric measurement of a low dissolved oxygen concentration.

### 3.5. Influence of the pH of the Electrolyte

**Figure 6:**
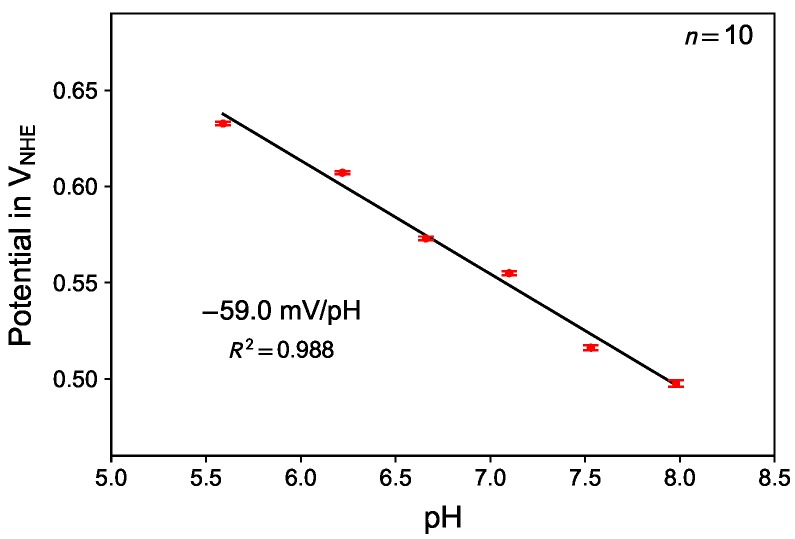
Influence of pH value: Measurement in air-saturated electrolyte with different pH. Potential is plotted with respect to normal hydrogen electrode (NHE).

Figure 6 shows the $E_{120s}$ values of the platinum electrode depending on the pH of the electrolyte in air-saturated electrolyte. The slope of the mean values from 10 measurements was found to be −59.0 mV/pH in case the results are expressed as potential with respect to normal hydrogen electrode (NHE). Correcting the obtained data by applying the concept of RHE, the data would show no change with pH.

This finding is in agreement with the assumption that both, the oxygen reduction and the platinum oxide formation process, shift with pH according to the Nernst equation (Equation (1)). This finding therefore justifies applying the potential scale of the RHE compensating the pH influence as it was done in all previous graphs.

## 4. Conclusions

Combining classical potentiometry with a prepolarization protocol, which we call *active potentiometry*, enables the measurement of dissolved oxygen concentration with potentiometric method at platinum electrodes. The logarithmic transfer function is especially helpful for biomedical applications with relevant concentration ranges over decades. The last potential of the active preconditioning before the open-circuit measurement determines the status of the electrode surface and therefore the condition during the potentiometric phase. Controlling the electrode surface is the main feature enabling reliable potentiometric oxygen measurements using platinum electrodes.

In contrast to copper, the oxide formation at platinum passivates the electrodes, and therefore the observed logarithmic transfer function cannot be interpreted as Nernstian behavior. The establishment of the potential can be described as a mixed potential based on currents occurring locally at the electrode only, assuming that no net current from the electrode flows into the electrometer amplifier. The electron donation process is the formation of platinum oxide, the electron accepting process is the reduction of molecular oxygen.

Platinum, with its excellent catalytic properties, is on one hand a good choice with respect to the oxygen reduction but comes along with weak selectivity towards other redox active substances, such as hydrogen peroxide. Therefore, we see the benefit of our approach especially in the application in Clark-type oxygen sensors [19,20], in which the measurement medium is separated from the sensor electrolyte using a gas-permeable membrane.

The described method is applicable to many different potentiometric sensors in which an active prepolarization can enhance sensitivity or selectivity for a specific parameter. An important feature is that no modification of the electrode setup itself is required, so the method can be employed to already existing sensors by enhancing the instrumentation hardware/software only.

## Abbreviations

The following abbreviations are used in this manuscript:

CV	Cyclic voltammogram
ML	Monolayer
NHE	Normal hydrogen electrode
OCP	Open circuit potential
PBS	Phosphate buffered saline
RHE	Reversible hydrogen electrode
